# Supplementary material for: Good quality end-of life care for people with an intellectual disability: A critical interpretive synthesis protocol
Source: PLoS One. 2024 Nov 8;19(11):e0311577. doi: 10.1371/journal.pone.0311577 (PMC11548725; doi:10.1371/journal.pone.0311577)
Supplement: S1 Table — (DOCX) [file pone.0311577.s001.docx]

**Data extraction form**

| **Title** |  | | |
| --- | --- | --- | --- |
| **Publication year** |  | | |
| **Author name/name of organisation** |  | | |
| **Country focus** | 🞎 High-income country(ies)  List specific countries:  🞎 Low- and middle-income country(ies)  List specific countries: | | |
| **Publication type** | 🞎Journal article  List specific journals:  🞎 Other literature | | |
| **Study design (if applicable)** | Primary research -  🞎 Systematic review  🞎 RCT  🞎 Cross-sectional  🞎 Cohort study  🞎 Interrupted time series  🞎 Before-after study  🞎 Qualitative study  🞎 Case study  🞎 Mixed methods  🞎 Other (specify)  Non-research -  🞎 Report  🞎 Thesis  🞎 Other review (i.e. not systematic)  🞎 Discussion/policy/position paper  🞎 Commentary/editorial/letter/correspondence  🞎 Website content  🞎 Guidance/guideline | | |
| **Type of participant and number**  (if applicable) | 🞎 People with an intellectual disability  🞎 Intellectual disability staff  🞎 Palliative care/hospice staff  🞎 Other health care professionals  🞎 Family caregivers  🞎 Other | | |
| **Method of data collection** | 🞎 Self complete questionnaire  🞎 Group interview  🞎 Individual interview  🞎 Focus group  🞎 Other | | |
| **Summary of key findings or insights from the document** |  | | |
| **Theme 1** | **Theme 2** | **Theme 3** | **Theme 4** |
| **Individual and**  **organisational commitment** | **Working together**  **in collaboration** | **The person’s story is at the heart of care** | **Developing tools and staff training** |
| Summary of information related to this theme: | Summary of information related to this theme: | Summary of information related to this theme: | Summary of information related to this theme: |
| **Additional information not captured in above themes:** | | | |
