# Supplementary material for: Good quality end-of life care for people with an intellectual disability: A critical interpretive synthesis protocol
Source: PLoS One. 2024 Nov 8;19(11):e0311577. doi: 10.1371/journal.pone.0311577 (PMC11548725; doi:10.1371/journal.pone.0311577)
Supplement: Attachment — Submitted filename: Response to reviewers.docx [file pone.0311577.s002.docx]

2^nd^ September 2024

Dear Dr Essouma,

We would like to thank you and other members of the editorial team for taking the time to review our manuscript. We appreciate your thoughtful comments and support. Our re-submitted manuscript reflects these comments and we have listed here our detailed responses to the points raised.

**Journal requirement 1**

Please ensure that your manuscript meets PLOS ONE's style requirements, including those for file naming.

**Response**:

The PLOS ONE style requirements have been considered and adhered to.

**Journal requirement 2**

In the online submission form, you indicated that [No datasets were generated or analysed during the current study. All relevant data from this study will be made available upon study completion.]. All PLOS journals now require all data underlying the findings described in their manuscript to be freely available to other researchers, either 1. In a public repository, 2. Within the manuscript itself, or 3. Uploaded as supplementary information.

This policy applies to all data except where public deposition would breach compliance with the protocol approved by your research ethics board. If your data cannot be made publicly available for ethical or legal reasons (e.g., public availability would compromise patient privacy), please explain your reasons on resubmission and your exemption request will be escalated for approval.

**Response**:

As this is a review protocol article, no dataset was generated or analyzed. All relevant data from the subsequent review will be made publicly available upon study completion.

**Additional Editor Comment 1**

**1a - Guidelines**

Consider conforming to PLOS ONE guidelines for formatting manuscripts and go to PLOS ONE website to look at published manuscripts in the journal.

**Response**:

The PLOS ONE style requirements have been considered and adhered to.

**1b - Abstract**

The abstract must be in one block without subheadings.

**Response**:

This has been amended as requested.

**1c – Protocol aim sub-section**

Consider deleting the “Protocol aim” sub-section and the information reported in that sub-section must be in the last paragraph of the introduction.

**Response**:

This sub-section has been deleted and the information incorporated in the last paragraph of the Introduction (lines 77-78 of the manuscript).

**1d - Keywords**

Consider including keywords in the manuscript after the abstract section. Those keywords should be among the important words in the title and the manuscript that will facilitate the retrieval of your future published paper online.

**Response**:

Keywords have now been inserted. Thank you for the suggestion.

**1e - References**

Format references according to the journal’s policy. Do you not see that there are way more references that would be expected in a protocol article?

**Response**:

The references have been formatted according to the journal’s policy. Following a revision of the manuscript, the number of references has been reduced.

**Additional Editor Comment 2 (Title)**

**2a – Title**

Are you going to keep “Good quality end of life care” or will you revise it to “High/Best/Optimal quality end of life care”? Because we usually talk of high-quality care, best-quality care, and optimal-quality care.

**Response**:

Thank you for this suggestion. After some consideration, we have decided to retain the existing title.

**2b - Definition**

Furthermore, you state in the introduction that the definition of “good end-of-life care” for this population group is still a matter of debate.

**Response**:

With the revision of the Introduction, this sentence has been removed.

**Additional Editor Comment 3 (Introduction)**

**3a – Length**

The current introduction section is very long and does not clearly help the reader understand why you are undertaking this study, especially because you will generate theoretical frameworks. The introduction needs to be reduced to max 1.5 pages (4 paragraphs max).

**Response**:

The length of the Introduction has been reduced as recommended.

**3b – Information contained in introduction**

I propose the report of these bits of information: what is the definition of intellectual disability? What is their best classification? Highlight the multiple health challenges encountered by individuals with intellectual disabilities (and their potential sociologic determinants such as diverse forms of inequalities/discrimination) that lead to their reduced quality of life and life expectancy compared to the general population. You could go further by saying that although their life expectancy appears to be lower than that of the general population, it is increasing, which could lead you to think more and more about their health, the quality of care they receive and their quality of life when they are adults and even elderly. Regarding quality of care, it would be more important to focus on the quality of end-of-life care to help them end their lives with dignity and respect.

**Response**:

The Introduction has been revised to reflect many of these topics. Thank you for your suggestions.

**3c – Research objectives**

Finally, you need to clearly state your research objectives.

**Response**:

The objective of the review has been added to the last paragraph of the Introduction (lines 75-77 of the manuscript).

**Additional Editor Comment 4 (Methods section)**

**4a – Background to Methods**

The information on lines 92-121 is confusing and somewhat unnecessary. I suggest revising that text by just stating that you will conduct and report this critical interpretive synthesis based on standard methods, and then you add current reference 30 which is indeed a good reference for the reporting of methods in this study. Then, by mentioning the different recommended sections of a critical interpretive synthesis article as sub-sections of the methods section in your article, unaware readers will understand by themselves how methods of a critical interpretive synthesis are reported. They will be able to find explanations in current reference 30.

**Response**:

These lines have been edited. Thank you for the suggestion.

**4b – Reporting framework**

This means I do not agree with your sentence on lines 122 and 123.

**Response**:

This sentence has been revised (lines 89-90 of the manuscript).

**4c – Sub-sections**

Consider conforming to reference 30 for writing the methods section of this article (please go through current reference 30 again to grasp the specifics needed in each sub-section), with the following methods’s sub-sections: searching the literature (information sources and search strategies), sampling (study selection), determination of quality (quality appraisal: conform to the quality appraisal of a critical interpretive synthesis, which is somewhat different from that of systematic reviews), data extraction, critical interpretive synthesis (reciprocal translational analysis, refutational synthesis, lines-of-argument synthesis).

**Response**:

Thank you for your thoughtful comments. We have revised these sub-sections in line with your recommendations.

**4d – Database searches**

Regarding the “searching literature” sub-section, … Why did you choose to only describe the search strategy in EMBASE? Why not for MEDLINE (PUBMED)? Could you report the different strategies in search databases as in this article (doi: 10.1371/journal.pone.0305112)?

**Response**:

Thank you for this suggestion. However we feel that for a protocol, it is sufficient to provide the search string to be used just for one database as an example. The different strategies to be adopted for all databases searched will be considered for inclusion in the review article with the results of each strategy adopted..

**4e – Search period**

What is the search period?

**Response**:

As per line 109 of the manuscript (‘eligibility criteria’), there are no restrictions on the timeframe.

**4f – CIS sub-section**

Please, make a thorough description of the critical interpretive synthesis sub-section. Propositions in the line-of-argument synthesis part of the critical interpretive synthesis sub-section should be based on your current knowledge about the quality of end-of-life care for people with intellectual disabilities which will be briefly highlighted. Go through current reference 30 and these two reviews for further inspiration https://doi.org/10.1016/j.contraception.2021.11.004 and <https://doi.org/10.1093/heapol/czae030>.?

**Response**:

We have expanded the sub-section which covers details of our approach to data analysis and synthesis. This includes reference to the ‘best-fit’ framework analysis that will be adopted to analyse the findings. Regarding your suggestion about items to be included in this sub-section, we do not feel it appropriate to include further details about the quality of end-of-life care for people with intellectual disabilities at this point in the protocol. This information has been provided in the Introduction section and will be discussed in detail in the subsequent review. Thank you for your recommendations for other reference material which we found useful.

**Additional Editor Comment 5 (Discussion section)**

**5a - Limitations**

Consider adding limitations statements. For example, your propositions in the lines-of-argument synthesis part of the CIS sub-section of the methods in this protocol article could be different from those in (or less elaborated than in) the final articles of the research as you may make new discoveries while conducting the study. This should be acknowledged herein.

**Response**:

A statement acknowledging a potential limitation of the CIS approach and how this will be addressed has been added to the Discussion section (lines 233-238 of the manuscript). This addresses the suggestion that CIS may lack transparency particularly in the generation of the synthesing argument. Measures to be put in place to mitigate against this potential limitation have also been included.

**5b – Dissemination Plan**

And delete the dissemination plan section. Rather mention how you plan to disseminate results of your research at the end of the abstract and the discussion section without explicitly stating that you are reporting the dissemination plan.

**Response**:

As recommended, the Dissemination plan section has been deleted and details included at the end of the Discussion section (lines 239-240 of the manuscript).

**Additional Editor Comment 6 (Conclusion)**

Consider adding a conclusion section after the discussion section.

**Response**:

A conclusion section has been added. Thank you for the suggestion.

**Additional Editor Comment 7 (Declarations)**

Declarations section should come after the conclusion section (except for the Funding statement and data availability statement which is required above as you did):

**Response**:

As per the Study Protocol Article Template, a Competing interests section is included at the start with the Funding statement and Data availability.

**Additional Editor Comment 7.1 (Funding)**

I am curious to know why your funder funded the study if they had no involvement in the research up to the decision to submit the article for publication.

**Response**:

The contribution of the Health Research Board (HRB) is noted because the IDS-TILDA study, which prompted this review and with which all authors are affiliated, is funded by the HRB. For clarity, this section has been revised to indicate support, rather than funding, by the HRB for this review.

**Additional Editor Comment 7.2 (Data availability statement)**

Did you want to state that no dataset was generated or analyzed for this protocol article?

**Response**:

The Data availability section has been amended (lines 17-18 of the manuscript). Thank you for the suggestion.

**Additional Editor Comment 7.3 (Conflict of interests)**

Where is the statement about conflicts of interests?

**Response**:

A Competing Interests section has now been added.

**Additional Editor Comment 7.4 (Acknowledgements)**

Acknowledgments not reported.

**Response**:

An Acknowledgements section has now been added.

**Additional Editor Comment 8 (Other)**

English language editing will be necessary so that the manuscript is as short as possible. I suggest deleting the figure in the current manuscript because I do not see its relevance.

**Response**:

The manuscript has been edited to ensure it is as short as possible. As suggested, the figure has been deleted.

Again, we very much appreciate the comments and recommendations we have received and thank you for your consideration. We hope that our re-submitted manuscript adequately addresses all points and we look forward to receiving further feedback in due course.

Yours sincerely,

Margaret Haigh
