# Supplementary material for: Good quality end-of life care for people with an intellectual disability: A critical interpretive synthesis protocol
Source: PLoS One. 2024 Nov 8;19(11):e0311577. doi: 10.1371/journal.pone.0311577 (PMC11548725; doi:10.1371/journal.pone.0311577)
Supplement: Attachment — Submitted filename: Response to reviewers 090924.docx [file pone.0311577.s003.docx]

11th September 2024

Dear Dr Essouma,

Thank you for taking the time to review our manuscript once again. We appreciate your thoughtful comments and support. Our re-submitted manuscript reflects your comments and we have listed here our detailed responses to the points raised.

**Journal requirement**

Please review your reference list to ensure that it is complete and correct. If you have cited papers that have been retracted, please include the rationale for doing so in the manuscript text, or remove these references and replace them with relevant current references. Any changes to the reference list should be mentioned in the rebuttal letter that accompanies your revised manuscript. If you need to cite a retracted article, indicate the article’s retracted status in the References list and also include a citation and full reference for the retraction notice.

**Response**:

The reference list has been checked to ensure it is complete and correct. Two references have been removed following the deletion of references to the (PRISMA-P) 2015 checklist (see point 7 below). The references which have been deleted are –

- Moher D, Shamseer L, Clarke M, Ghersi D, Liberati A, Petticrew M, et al. Preferred reporting items for systematic review and meta-analysis protocols (PRISMA-P) 2015 statement. Syst. Rev. 2015;4(1):1. doi: 10.1186/2046-4053-4-1
- Shamseer L, Moher D, Clarke M, Ghersi D, Liberati A, Petticrew M, et al. Preferred reporting items for systematic review and meta-analysis protocols (PRISMA-P) 2015: elaboration and explanation. BMJ. 2015;350:g7647. doi: 10.1136/bmj.g7647

**Additional Editor Comments**

1. You have already stated in the introduction and at the beginning of the methods section that this is the protocol of a critical interpretive synthesis. You do not need to repeat in all sub-sections of the methods section and elsewhere in the manuscript "in a critical interpretive synthesis" and what a criticial interpretive synthesis is about. For example, the sentence on lines 172 to 174 is unnecessary.

Response:

Thank you for this observation. The lines identified have now been deleted. In addition, amendments have been made to the following sub-sections to reduce repetition while still framing the approach within a CIS context – Eligibility, Determination of quality, Data extraction, Data analysis and synthesis and Discussion.

2. Avoid bullet as much as possible in the text and make sentences. Example on lines 182 to 185.

Response:

Bullets have been removed from the lines suggested. However, it was decided to retain bullets for the inclusion and exclusion criteria for clarity.

3. Along this line, reduce the text under the data analysis sub-section of the methods section with only information about how you will synthesize and interpret data in your review.

Response:

This text has now been reduced to reflect the process of how the research team will go about synthesizing and interpreting the data in the review.

4. Please, can you add links of websites where references 5, 13 and 16 can be found?

Response:

These have now been added.

5. Line 49: I would say “It was” because the systematic review suggesting 1% was published in 2011 and the frequency may actually have changed.

Response:

Thank you for this suggestion. This has been amended accordingly

6. Lines 81-83: “This decision was informed by the findings of an initial review of the literature in relation to the end-of-life experiences of people with an intellectual disability which established that the body of evidence is similarly diverse and complex.” The reference supporting this statement (of the corresponding systematic review) is needed.

Response:

This was a preliminary review carried out by the lead author and involved semi-structured searches of electronic databases. Discussions were also held with members of the research team who are acknowledged experts in this field and who vindicated the findings of the preliminary review.

7. Lines 89 and 90: “This protocol will use the Preferred Reporting Items for Systematic 89 Review and Meta-Analysis Protocols (PRISMA-P) 2015 checklist [19, 20] (S1 Table).” Consider deleting this sentence and S1 Table because this is not the protocol of a systematic review whilst PRISMA-P guides reporting of systematic review protocols.

Response:

Thank you for this suggestion. These lines referring to the PRISMA-P checklist and supporting table have now been removed.

8. Under the "Searching the literature" sub-section of the Methods section, consider presenting the search strategy (specifying the search period as well) before eligibility criteria, and then literature screening.

Response:

Thank you for this suggestion which we have considered and have partly adhered to. We would prefer to keep the Eligibility Criteria sub-section, with the inclusion criteria, where it is currently placed because it was in identifying the inclusion criteria that we obtained clarity to help us develop the search strategy. The exclusion criteria (which have been revised in line with your suggestions below) have been moved to the Literature Screening section as it will be at this point that the exclusion criteria, as now defined, will be referred to. We hope that the reasons for our amendments are clear.

9. Consider revising the eligibility criteria. Inclusion criteria would best be framed indicating study designs ( I guess you will find quantitative observational and interventional studies, qualitative and mixed-methods studies) that will be included as well as using the PICO (Population [this should be only individuals with intellectual disability, not care providers as well as you report in the present manuscript], Intervention [End-of-life care what specification of what you mean by end-of-life care as I mentioned in the last round of review], Comparator [Individuals without intellectual disabilities for studies using a comparator group], and Outcome [clearly define the outcomes you will be looking for: quality of life...]. Exclusion criteria are not teh contrary of inclusion criteria, but criteria that led to the exclusion of studies after their initial fulfilment of inclusion criteria.

Response:

The eligibility criteria sub-section has been reviewed and revised as outlined below. In relation to your specific points raised -

- As all study designs will be considered, the criteria for this are defined as ‘any’. Non-empirical literature will also be considered.
- As noted earlier, this is not a systematic review and therefore the PICO model was not strictly adhered to in the development of the search strategy. Other than comparator, which is not relevant in this review, all elements have been considered in the development of the search strategy.
- In terms of our population, it is vital that studies involving care providers are included because much of the research in the field of intellectual disability is informed by this cohort and not by people with intellectual disability.
- We have purposively not made a specification about end-of-life care as, in line with CIS methodology, we have opted to have less well-defined boundaries when searching for suitable literature. It will be at the quality appraisal stage that we will apply a critical approach to determine inclusion.
- The outcome is referred to in the ‘content’ in the inclusion criteria. Here, it is specified that to be included the literature must contain reference to care at end-of-life for people with an intellectual disability.
- We have revised the exclusion criteria as per your recommendation. Thank you for this clarification. As outlined in our response to point 8 above, the exclusion criteria have been moved to the Literature Screening section. We hope that this meets with your approval.

10. Line 139: did you want to say "Any uncertainty regarding study inclusion"?

Response:

This has been amended accordingly

11. Line 143: low threshold of what: study quality? The quote should be removed on eligible.

Response:

This sentence has been revised to ‘a high tolerance threshold will be applied ….’ as we want to convey an impression on inclusivity to the reader.

The quote has been removed from the word ‘eligible’.

12. The questions on lines 150 to 155 should be on the same line following the text on line 149.

Response:

This has been amended as suggested.

13. Is there a reference for the criteria listed on lines 148 to 155? Consider adding it.

Response:

References are included in the preceding lines.

14. Altogether, there is some confusion about how you will determine the quality of studies to include in your review, making it necessary to clearly revise the text on lines 143 to 162.

Response:

The ‘Determination of quality’ section has been revised to reflect your comment and we hope the revised text is clearer.

15. Consider revising the text under the data extraction sub-section reducing its length with only the information about the data that will be extracted (consider extracting data about the study [design, author, location, method of data collection, study period] and PICO elements because those are elements that will help you meet your review goals), how you will extract those data and how you will resolve discrepancies arising between people who will extract data.

Response:

The Data extraction sub-section has been revised as suggested and is in line with the proposed procedure for this stage in the process. The data extraction form has also been updated.

16. Line 224: is the word "complex" necessary here since everything can be considered to be complex?

Response:

This word has been removed

17. Lines 225 to 227: "The approach will entail adopting procedures of conventional systematic review methodology to search and review the literature, combined with interpretive synthesis associated with qualitative research." I do not agree with this sentence. Consider sticking to the critical interpretive synthesis although we know that this type of review incorporates some elements of the systematic. It is at the beginning of the methods section that you should showcase how critical interpretive synthesis incorporates elements of the systematic review and not repeat that description elsewhere in the manuscript. If you really want to extensively describe how a critical interpretive synthesis compares with a systematic review, you could do that in a table that you append to the text at the beginning of the methods section.

Response:

These lines have now been removed from the Discussion section and we have amended the beginning of the Methods section slightly to align with your suggestion. We hope that the current version is satisfactory.

18. Why has one author been added? And the reasons of changes in authors positions should be clearly mentioned.

Response:

This author has been added because she will be involved in reviewing the material for inclusion in the review and, as a result, has reviewed this manuscript. On reflection, it has been decided to move her name to later in the list of authors to reflect her contribution to the manuscript.

Again, we very much appreciate the comments and recommendations we have received and thank you for your consideration. We hope that our re-submitted manuscript adequately addresses all points and we look forward to receiving further feedback in due course.

Yours sincerely,

Margaret Haigh
